# Annatto-extracted tocotrienols improve glucose homeostasis and bone properties in high-fat diet-induced type 2 diabetic mice by decreasing the inflammatory response

**DOI:** 10.1038/s41598-018-29063-9

**Published:** 2018-07-27

**Authors:** Chwan-Li Shen, Gurvinder Kaur, Desiree Wanders, Shaligram Sharma, Michael D. Tomison, Latha Ramalingam, Eunhee Chung, Naima Moustaid-Moussa, Huanbiao Mo, Jannette M. Dufour

**Affiliations:** 1 Department of Pathology, Texas Tech University Health Sciences Center, Lubbock, TX USA; 2 Department of Cell Biology and Biochemistry, Texas Tech University Health Sciences Center, Lubbock, TX USA; 3 Department of Nutrition, Georgia State University, Atlanta, GA USA; 4 Department of Nutritional Sciences, Texas Tech University, Lubbock, TX USA; 5 Department of Kinesiology, Health, and Nutrition, University of Texas at San Antonio, San Antonio, TX USA; 6 Obesity Research Cluster, Texas Tech University, Lubbock, TX USA

## Abstract

Diabetes is a risk factor for osteoporosis. Annatto-extracted tocotrienols (TT) have proven benefits in preserving bone matrix. Here, we evaluated the effects of dietary TT on glucose homeostasis, bone properties, and liver pro-inflammatory mRNA expression in high-fat diet (HFD)-induced type 2 diabetic (T2DM) mice. 58 male C57BL/6 J mice were divided into 5 groups: low-fat diet (LFD), HFD, HFD + 400 mgTT/kg diet (T400), HFD + 1600 mgTT/kg diet (T1600), and HFD + 200 mg metformin/kg (Met) for 14 weeks. Relative to the HFD group, both TT-supplemented groups (1) improved glucose homeostasis by lowering the area under the curve for both glucose tolerance and insulin tolerance tests, (2) increased serum procollagen I intact N-terminal propeptide (bone formation) level, trabecular bone volume/total volume, trabecular number, connectivity density, and cortical thickness, (3) decreased collagen type 1 cross-linked C-telopeptide (bone resorption) levels, trabecular separation, and structure model index, and (4) suppressed liver mRNA levels of inflammation markers including IL-2, IL-23, IFN-γ, MCP-1, TNF-α, ITGAX and F4/80. There were no differences in glucose homeostasis and liver mRNA expression among T400, T1600, and Met. The order of osteo-protective effects was LFD ≥T1600 ≥T400 = Met >HFD. Collectively, these data suggest that TT exerts osteo-protective effects in T2DM mice by regulating glucose homeostasis and suppressing inflammation.

## Introduction

Diabetes mellitus (DM) is a chronic metabolic disorder that affects more than 30.3 million people in the United States; an additional 84.1 million individuals have been diagnosed with prediabetes^1^. DM with long-term hyperglycemia may have a direct, adverse impact on bone and the bone marrow microenvironment including (i) advanced glycation end products of bone matrix proteins^2^, (ii) chronic inflammation and abnormal cytokine and adipokine production, resulting in detrimental effects on bone cells, (iii) impaired neuromuscular/skeletal interactions^3,4^, and (iv) excessive production of reactive oxygen species^5^. Mediated by the alterations of these enzymatic and non-enzymatic events, DM induces crosslinking negative effects including significant bone loss, poor bone quality and impaired biomechanical properties in spontaneously diabetic WBN/Kob rats^6^. Animal studies in streptozotocin (STZ)-induced DM rats have confirmed that a relationship exists between type 1 DM and poor bone quality^7^. In humans, Miyaka *et al*. recently showed the association of reduced bone mineral density and several vertebral fractures with increased mortality in people with type 2 DM (T2DM)^8^. A recent meta-analysis confirmed that there was an increased prevalence of low bone mass-related fractures in people with T2DM compared to those withoutT2DM^9^.

T2DM is characterized by peripheral insulin resistance with a variable degree of hyperglycemia and impaired insulin secretion after metabolic challenge by glucose^10^. In T2DM, chronic oxidative stress and inflammation, resulting from enhanced exposure to oxidants, can lead to insulin resistance and impaired insulin signaling and glucose transport in skeletal muscle^11^. Oxidative stress and chronic inflammation have detrimental effects on skeletal muscle, pancreatic beta cells, and bone^2,12,13^, causing imbalance in bone metabolism and bone loss^2,12–14^.

The use of dietary supplements, botanicals, and nutraceuticals has become an alternative approach to prevent and mitigate the complications of hyperglycemia in people with T2DM, including alleviating inflammation and maintaining the oxidant-antioxidant balance^15^. Vitamin E is a collective term for tocopherols and tocotrienols (TT)^16^. Both tocopherols and TT exist in 4 different methyl substitutions in nature: alpha (α), beta (β), gamma (γ), and delta (δ) consisting of a mixture of varying compositions^16^. Most plant species contain tocopherols, whereas TT can only be found in certain plants such as annatto, palm, grains, and nuts^17^. Preclinical studies have shown the effect of a mixture of TT on glucose homeostasis in STZ-treated type 1 DM animals through down-regulation of transforming growth factor-β^18^ or suppression of oxidative stress^19^. Vafa *et al*. further reported that supplementation of TT-enriched canola oil reduced fasting blood sugar levels and lipid oxidation, while increasing total antioxidant capacity in T2DM patients^20^. With regard to bone aspect, several animal studies using δ-TT^21^, γ-TT^22^, palm oil-extracted TT^23^, and annatto-extracted TT^24^ in sex hormone-deficient animal models all showed the osteo-protective effects of TT in terms of bone matrix and microstructure. However, no study has yet evaluated the beneficial effects of TT on bone properties in animals with T2DM.

With its strong anti-oxidative/anti-inflammatory properties, annatto-extracted TT, consisting of 90% δ -TT and 10% γ -TT, would be a good candidate to evaluate TT’s effects on T2DM-related complications, such as hyperglycemia and bone deterioration. We previously reported that δ-TT inhibited adipogenesis in 3T3-F442A preadipocytes^25^ and increased fatty acid oxidation, reduced adipocyte hypertrophy, and suppressed inflammation in both liver and adipose tissue^26^. In this paper, we focused on the beneficial effect of annatto-extracted TT on glucose homeostasis and bone health in a high-fat diet (HFD)-induced T2DM model. We hypothesized that annatto-extracted TT has both anti-diabetic and anti-osteoporotic properties in HFD-induced T2DM animals through suppression of inflammation.

## Results

### Body weight, feed intake, and water consumption

**Figure 1:**
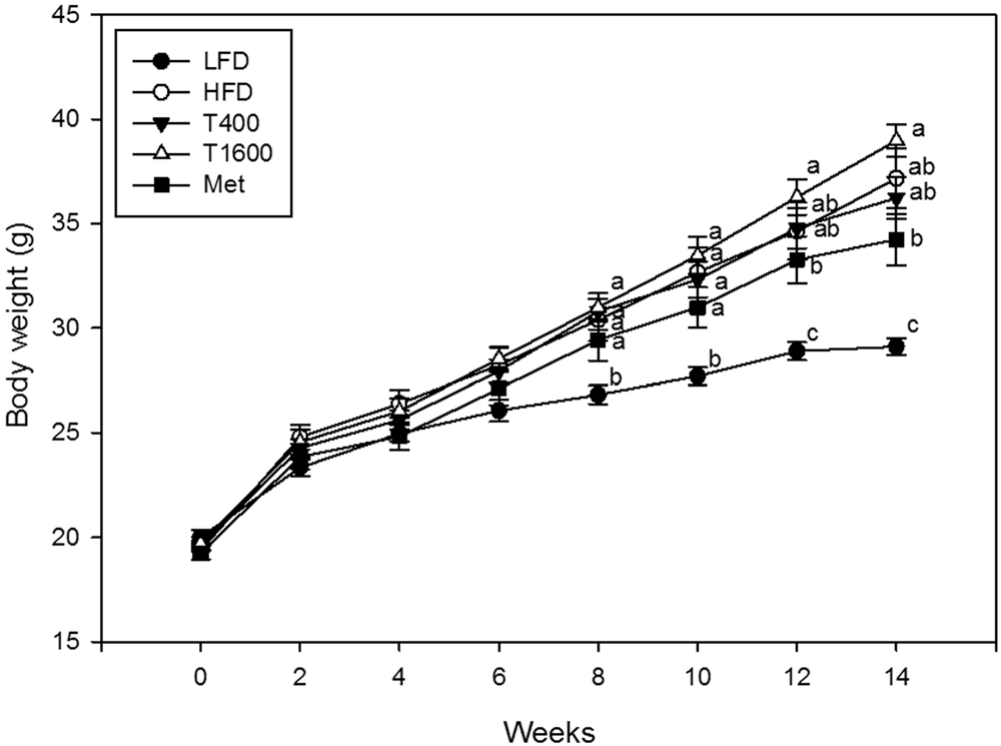
Effect of annatto-extracted tocotrienols on body weight. LFD, low-fat diet; HFD, high-fat diet; T400, HFD + TT at 400 mg/kg diet; T1600, HFD + TT at 1600 mg/kg diet; and Met, HFD + metformin at 200 mg/kg diet. Values at the same week not sharing a common letter are statistically different (p < 0.05. n = 8–10).

Figure 1 presents the mean body weight for different treatment groups. At baseline, there was no significant difference in body weight among all groups. Throughout the study period, body weight significantly increased in all animals regardless of treatment. From 8 weeks to the end of study, the body weight significantly increased in all HFD-fed treatment groups relative to the low-fat diet (LFD) group. At both 12 and 14 weeks, there was no significant difference in body weight among the HFD, T400, and T1600 groups. At the end of the 14-week study, the statistical results showed the order of body weight as follows: T1600 group = HFD group = T400 group >metformin (Met) group >LFD group (p < 0.001). Throughout the study, the average food intake by weight and water consumption were similar among all groups (both p > 0.05).

### Glucose homeostasis and morphological analysis of the pancreas

**Figure 2:**
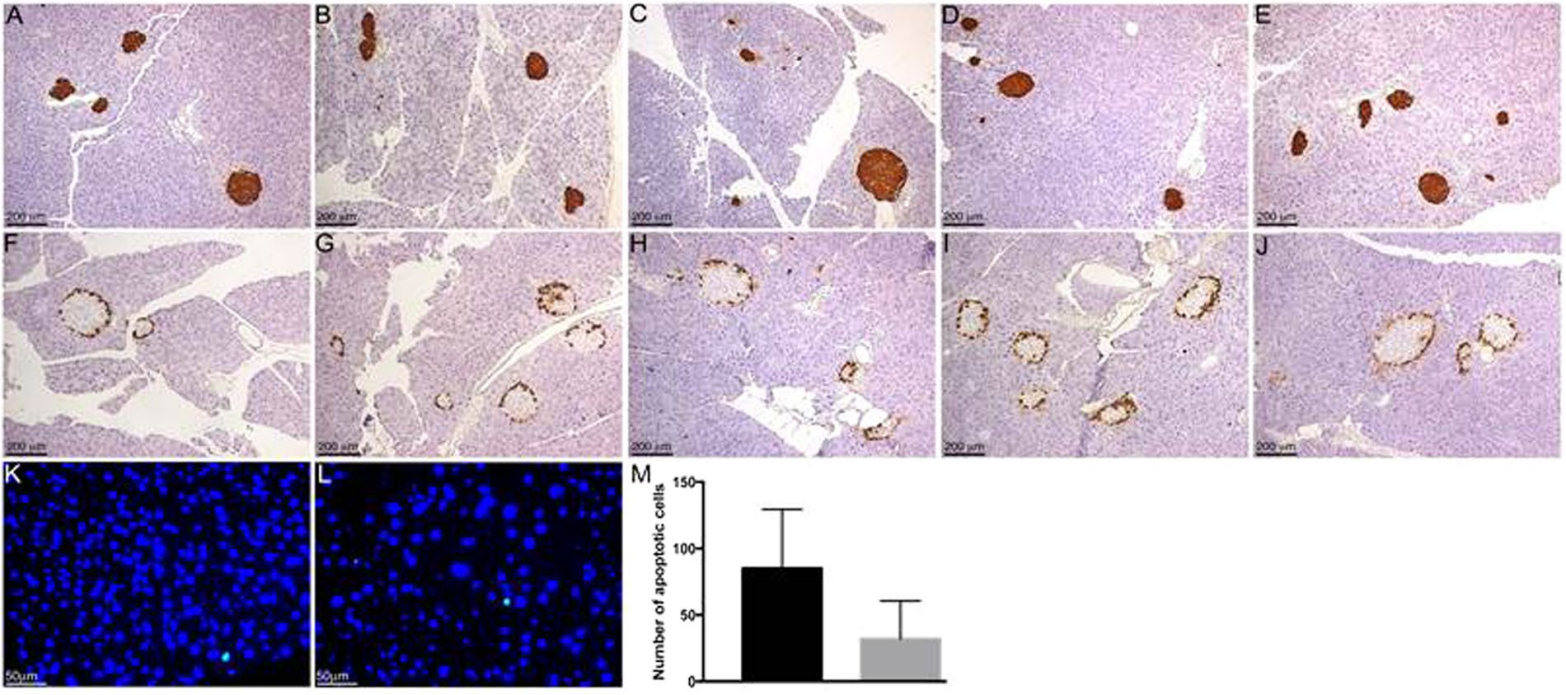
Effect of annatto-extracted tocotrienols on immunohistochemical and TUNEL analysis of pancreatic tissue. Pancreatic tissue sections collected from mice fed LFD (**A** and **F**), HFD (**B** and **G**), and HFD supplemented with T400 (**C** and **H**), T1600 (**D** and **I**) or Met (**E** and **J**) were immunostained for insulin (**A**–**E**) or glucagon (**F**–**J**) and counterstained with hematoxylin. Pancreatic tissue sections collected from mice fed LDF (**K**) or HFD (**L**) were analyzed for apoptosis by TUNEL assay (green color, **K** and **L**). Sections were counterstained with DAPI (blue color, **K** and **L**). Total numbers of apoptotic cells from LFD (grey bar) or HFD (black bar) groups (**M**) were shown. Data shown are means ± SEM (n = 4). Significant differences in means were determined by the unpaired Student t-test method at a P value of ≤0.05.

The effects of TT supplementation on glucose homeostasis (Table 1) were assessed by glucose tolerance test (GTT) and insulin tolerance test (ITT), serum and pancreatic insulin levels^27^, and pancreatic histology (Fig. 2). Relative to the LFD group, the HFD group exhibited hyperglycemia after fasting and glucose intolerance indicative of diabetes (Table 1). Supplementation with T1600 and Met resulted in a significant decrease in 4-hour fasting blood glucose levels. The T400, T1600 and Met groups all had improved glucose tolerance when compared to the HFD group. No significant difference in GTT area under curve (AUC) was observed among the T400, T1600, and Met groups.

**Table 1:** Effect of annatto-extracted tocotrienol supplementation on glucose homeostasis^a^.

Groups	Time (min)					AUC
	0	15	30	60	120	
***GTT***						
LFD	150.4^b^ ± 8.8	408.9 ± 23.8	409.8^b^ ± 26.5	227.5^c^ ± 12.9	160.6^d^ ± 8.9	31541.6^c^ ± 1455.7
HFD	186.3^a^ ± 10.6	506.2 ± 24.5	542.0^a^ ± 16.9	512.3^a^ ± 37.8	369.3^a^ ± 22.6	55486.5^a^ ± 2522.1
T400	163.3^ab^ ± 7.5	478.9 ± 24.0	512.1^a^ ± 19.1	408.8^b^ ± 30.0	285.1^b^ ± 20.5	46889.0^b^ ± 1710.9
T1600	155.5^b^ ± 9.1	473.7 ± 22.9	517.5^a^ ± 24.9	428.0^b^ ± 12.2	228.8^c^ ± 18.9	46041.5^b^ ± 1626.2
Met	148.5^b^ ± 5.5	485.2 ± 29.8	547.2^a^ ± 15.3	423.5^b^ ± 16.2	257.3^bc^ ± 12.6	47479.5^b^ ± 1154.2
***ITT***						
LFD	138.3^b^ ± 4.7	81.2 ± 6.4	68.3^b^ ± 4.9	31.8^b^ ± 3.8	33.6^c^ ± 3.5	5621.2^c^ ± 245.1
HFD	171.2^a^ ± 6.9	88.6 ± 7.1	81.8^b^ ± 3.3	68.6^a^ ± 3.6	90.8^a^ ± 3.5	9313.5^ab^ ± 301.1
T400	166.5^a^ ± 4.9	100.3 ± 13.6	77.6^b^ ± 4.9	63.8^a^ ± 4.1	79.2^ab^ ± 7.1	8840.2^ab^ ± 579.8
T1600	168.1^a^ ± 6.5	91.5 ± 6.9	96.0^a^ ± 6.0	52.5^b^ ± 8.0	72.0^b^ ± 4.3	8364.6^b^ ± 279.4
Met	180.3^a^ ± 8.2	96.3 ± 6.7	96.5^a^ ± 2.5	71.3^a^ ± 4.1	83.2^ab^ ± 5.9	9619.6^a^ ± 355.3

^a^Values are mean ± SEM, n ≥ 6. Within the same column, values not sharing a common letter are statistically different (p < 0.05).

LFD, low-fat diet; HFD, high-fat diet; T400, HFD + TT at 400 mg TT/kg diet; TT1600, HFD + TT at 1600 mg TT/kg diet; Met, HFD + metformin at 200 mg metformin/kg diet; AUC, area under the curve; GTT, intra-peritoneal glucose tolerance test; ITT, intra-peritoneal insulin tolerance test.

The HFD group developed insulin resistance as shown by a slower lowering of blood glucose levels after insulin injection and a greater ITT AUC compared to the LFD group (Table 1). There was no significant difference in ITT AUC among the HFD, T400 and Met groups. Clearly, the T1600 group had lower ITT AUC than that of the Met group (Table 1).

There was no significant difference in serum or pancreatic insulin among all groups, as shown in our previous paper^26^. Consistent with the serum and pancreatic insulin levels, there were no differences in immunostaining of insulin (Fig. 2A,B), marker for islet beta cells, or glucagon (Fig. 2F,G)), marker for islet alpha cells, in the pancreas between LFD (Fig. 2A,F) and HFD (Fig. 2B,G) groups. In addition, there were no major differences among T400, T1600, and Met groups (Fig. 2C–E and H–J) or between LFD and HFD groups. TUNEL analysis of apoptosis in pancreatic cells also revealed no significant difference between LFD and HFD groups (Fig. 2K–M).

### Serum bone turnover markers

**Figure 3:**
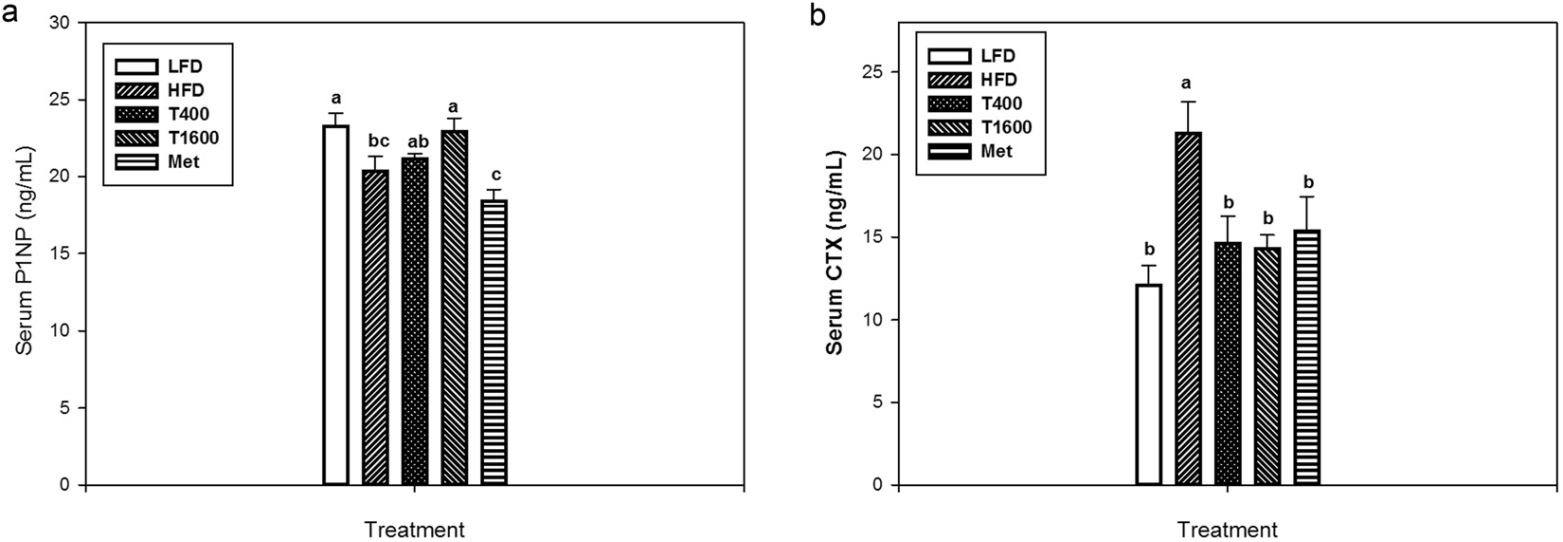
Effect of annatto-extracted tocotrienols on P1NP (**a**) and CTX (**b**). LFD, low-fat diet; HFD, high-fat diet; T400, HFD + TT at 400 mg/kg diet; T1600, HFD + TT at 1600 mg/kg diet; and Met, HFD + metformin at 200 mg/kg diet. CTX, c-terminal telopeptide; P1NP, procollagen I intact N-terminal. Within the same figure, values not sharing a common letter are statistically different (p < 0.05). n = 8–10.

Figure 3 shows the effects of TT supplementation on serum P1NP, a serum bone formation biomarker (Fig. 3a) and collagen type 1 cross-linked C-telopeptide (CTX), a serum bone resorption biomarker (Fig. 3b). Relative to LFD group, the HFD group had decreased serum procollagen I intact N-terminal propeptid (P1NP) levels. TT supplementation increased serum P1NP, but only the T1600 group had a significantly higher P1NP level than that of the HFD group. There was no difference in serum P1NP level among the LFD, T400, and T1600 groups. Furthermore, the Met group had the lowest serum P1NP concentrations among all treatment groups. The HFD group had an increased serum CTX than the LFD group, an effect that was reversed in T400, T1600, and Met groups. There was no significant difference in CTX among the LFD, T400, T1600, and Met groups.

### Microarchitectural properties of LV-4 and femur

Table 2 exhibits the effects of TT supplementation on trabecular microarchitecture at lumbar vertebrae-4 (LV-4) and the femur. As expected, the HFD adversely affected 3-dimensional bone microstructure. Compared to the LFD group, HFD significantly decreased LV-4 and femoral trabecular bone volume/total volume (BV/TV), trabecular number (Tb.N), and connectivity density (Conn.Dn), increased trabecular separation (Tb.Sp) and structure model index (SMI), and had no effect on trabecular thickness (Tb.Th). TT supplementation (both T400 and T1600) mitigated the HFD-induced deterioration of trabecular bone microstructure as shown by increases in BV/TV, Tb.N, and Conn.Dn and decreases in Tb.Sp and SMI at both LV-4 and femur. The osteo-protective impacts of T1600 treatment on these trabecular bone microstructures (e.g., LV-4 SMI and all distal femur trabecular bone parameters) of obese mice are greater than those of T400 treatment, resulting in no difference between the T1600 and the LFD group.

**Table 2:** Effect of annatto-extracted tocotrienol supplementation on bone microstructure properties of LV-4 and femur^a^.

Variables	LFD	HFD	T400	T1600	Met	P value
*LV-4 (trabecular bone)*						
BV/TV, %	19.88^a^ ± 0.58	16.96^b^ ± 0.32	17.72^b^ ± 0.69	18.72^ab^ ± 0.63	17.18^b^ ± 0.94	0.022
Tb.N, mm^−1^	5.61^a^ ± 0.06	5.35^b^ ± 0.03	5.36^b^ ± 0.07	5.47^ab^ ± 0.09	5.30^b^ ± 0.05	0.011
Tb.Th, mm	0.045 ± 0.008	0.045 ± 0.001	0.045 ± 0.001	0.044 ± 0.001	0.046 ± 0.001	0.934
Tb.Sp, mm	0.175^b^ ± 0.002	0.186^a^ ± 0.001	0.183^ab^ ± 0.002	0.177^b^ ± 0.003	0.187^a^ ± 0.001	<0.001
Conn.Dn, mm^−3^	213.0^a^ ± 15.3	161.5^c^ ± 8.3	177.0^bc^ ± 8.0	204.4^ab^ ± 13.3	158.3^c^ ± 8.3	0.002
SMI	1.363^b^ ± 0.110	1.749^a^ ± 0.091	1.715^a^ ± 0.095	1.379^b^ ± 0.089	1.867^a^ ± 0.120	0.002
*Distal femur (trabecular bone)*						
BV/TV, %	9.35^ab^ ± 0.61	5.86^c^ ± 0.35	6.95^bc^ ± 0.80	10.91^a^ ± 1.34	7.80^bc^ ± 1.67	0.010
Tb.N, mm^−1^	4.03^a^ ± 0.15	3.29^b^ ± 0.10	3.40^b^ ± 0.12	3.86^a^ ± 0.12	3.25^b^ ± 0.15	<0.001
Tb.Th, mm	0.049 ± 0.001	0.047 ± 0.001	0.052 ± 0.002	0.054 ± 0.002	0.050 ± 0.003	0.208
Tb.Sp, mm	0.247^b^ ± 0.008	0.307^a^ ± 0.009	0.297^a^ ± 0.010	0.259^b^ ± 0.009	0.316^a^ ± 0.015	<0.001
Conn.Dn, mm^−3^	50.59^a^ ± 5.93	22.46^b^ ± 3.49	27.60^b^ ± 6.30	51.23^a^ ± 9.96	24.51^b^ ± 7.93	0.005
SMI	2.729^bc^ ± 0.051	3.063^a^ ± 0.091	2.898^ab^ ± 0.123	2.470^c^ ± 0.146	3.027^ab^ ± 0.169	0.007
*Femur mid-diaphysis (cortical bone)*						
B.Ar, mm^2^	0.95 ± 0.02	0.89 ± 0.03	0.89 ± 0.02	0.93 ± 0.03	0.90 ± 0.04	0.440
T.Ar, mm^2^	2.07 ± 0.04	1.99 ± 0.04	2.01 ± 0.06	2.07 ± 0.05	1.95 ± 0.04	0.369
Ct.Th, mm	0.221^a^ ± 0.003	0.208^b^ ± 0.004	0.223^a^ ± 0.004	0.222^a^ ± 0.005	0.204^b^ ± 0.006	0.019

^a^Values are mean ± SEM, n = 10. Within the same row, values not sharing a common letter are statistically different (p < 0.05).

B.Pm, bone perimeter; BV/TV, bone volume/total volume; B.Ar, cross-sectional bone area; Conn.Dn, connectivity density; Ct.Th; cortical thickness; HFD, high-fat diet; LFD, low-fat diet, LV-4, lumbar vertebrae-4; Met, HFD + metformin at 200 mg/kg diet; T400, HFD + TT at 400 mg/kg diet; TT1600, HFD + TT at 1600 mg/kg diet; T.Ar, cross-sectional total area; Tb.N, trabecular number; Tb.Pf, trabecular bone pattern factor; Tb.Sp, trabecular separation; Tb.Th, trabecular thickness; SMI, structure model index.

Regarding femoral midshaft bone microstructure, no significant difference in cross-sectional bone area (B.Ar) and total area (T.Ar) was observed between any treatment groups. TT supplementation (both T400 and T1600) prevented the obesity-induced loss of femoral cortical thickness (Ct.Th) and restored it to the level of LFD group (LFD = T400 = T1600). Treating obese mice with metformin did not improve any trabecular or cortical microstructures in obese mice, as shown by no significant difference in any of the bone microstructure parameters between the HFD group and the Met group.

### Effects on liver mRNA expression

Relative to the LFD group, the HFD group significantly upregulated liver mRNA expression of pro-inflammatory cytokines including interleukin (IL)-2, IL-23, interferon (IFN)-γ, monocyte chemoattractant protein (MCP)-1, and monocyte/macrophage markers such as ITGAX, and F4/80 (Table 3). The levels of hepatic mRNA of the pro-inflammatory cytokines and monocyte/macrophage markers in the T400, T1600 and Met groups were significantly lower than those of the HFD group, but not different from those of the LFD group.

**Table 3:** Effect of annatto-extracted tocotrienol supplementation on liver mRNA expression.

Variables (Target, x10^3^/cyclophilin)	LFD	HFD	T400	T1600	Met	P value
IL-2	2.00^b^ ± 0.43	12.70^a^ ± 4.00	1.91^b^ ± 0.27	2.22^b^ ± 0.30	2.15^b^ ± 0.27	<0.001
IL-23	9.40^b^ ± 1.61	25.50^a^ ± 9.79	6.39^b^ ± 1.21	7.66^b^ ± 1.42	5.68^b^ ± 0.89	0.023
IFN-γ	6.14^b^ ± 1.47	28.50^a^ ± 10.70	6.85^b^ ± 1.43	8.59^b^ ± 2.46	8.18^b^ ± 2.44	0.013
MCP-1	12.10^b^ ± 1.66	34.10^a^ ± 7.07	13.20^b^ ± 1.59	13.30^b^ ± 2.46	15.70^b^ ± 7.06	0.016
ITGAX	0.92^b^ ± 0.21	2.89^a^ ± 1.06	1.05^b^ ± 0.19	1.28^b^ ± 0.29	1.17^b^ ± 0.23	0.028
F4/80	5.94^b^ ± 0.82	12.70^a^ ± 3.28	5.19^b^ ± 1.08	5.41^b^ ± 1.23	5.71^b^ ± 0.88	0.014

Data are mean ± SEM. Within the same row, values not sharing a common letter are statistically different (p < 0.05). IFN-γ, interferon-γ; IL, interleukin; ITGAX, integrin subunit alpha x; MCP-1, monocyte chemoattractant protein-1.

## Discussion

In this study, we employed a HFD-induced obese T2DM rodent model to examine the effects of TT supplementation on glucose homeostasis and bone properties and underlying molecular mechanisms in male mice. To the best of our knowledge, this is the first *in vivo* study to demonstrate the efficacy of annatto-extracted TT on osteo-protection in an obese T2DM model, possibly via the suppression of inflammation and regulation of glucose homeostasis.

Consistent with previous studies^28^, HFD feeding produced a detrimental effect on bone matrix and bone microarchitecture indicated by lowered trabecular volume fraction, number, and Conn. Dn, and increased trabecular separation and SMI in LV-4 and femur (Table 2). Importantly, TT conserved bone matrix and protected bone microstructure from deterioration in HFD male mice, which supports our hypothesis that TT increased BV/TV through restoring bone microstructure (an increase in Tb.N and SMI as well as a decrease in Tb.Sp and Conn.Dn). In addition, as shown in Fig. 3, TT supplementation in the diet mitigated bone deterioration in HFD-fed mice by enhancing bone formation (as shown by higher P1NP) and suppressing bone resorption (as shown by lower serum CTX). The levels of P1NP and CTX indicated a higher ratio of bone formation to resorption that benefits bone remodeling in annatto-TT-supplemented groups. Such osteo-protective effects of TT are in agreement with previous studies feeding δ-TT to ovariectomized rats^21^, γ-TT to ovariectomized mice^22^ and growing male rats^29^, palm oil-extracted TT to ovariectomized rats^23^, and annatto-TT to orchidectomized rats^24^. The ability of TT to favor bone remodeling demonstrated in the present study maybe attributable to TT’s effect on osteoblasts^30^ and osteoclasts^27,31^. Nizar *et al*.^30^ has demonstrated that γ-TT at low concentration protects osteoblasts against oxidative stress due to its anti-oxidative activity, whereas Brooks *et al*. reported γ-TT inhibited osteoclast formation and activity^31^. Ha *et al*.^27^ further demonstrated that δ-TT, but not tocopherol, inhibits the activation of receptor activator of nuclear factor-κB ligand (RANKL) and extracellular signal-regulated kinase (ERK) and consequently, osteoclast differentiation and activation, offering anti-bone resorptive properties.

In addition to the direct effects of TT on bone health, another benefit of TT in our model is prevention of diabetes. One likely mechanism driving osteoporosis in the T2DM population is impaired glucose homeostasis. For example, hyperglycemia can cause an increase in differentiation of bone marrow mesenchymal cells into adipocytes, which in turn could increase adipogenesis and attenuate osteogenesis as a result of suppression of osteoblast function and number, gene expression, and maturation^32^. While hyperglycemia, elevated serum triglycerides and insulin resistance were observed in the HFD group, supplementation with TT for 14 weeks significantly improved overall glucose homeostasis by decreasing fasting blood glucose level and improving glucose and insulin tolerances (Table 1). Our findings agree with previous studies in which TT-rich fractions from palm or rice bran oils significantly improved glycemic status in diabetic rats^18,19^. In addition, our results also are in the agreement with Wong’s finding^33^ that δ-TT improved glucose tolerance and insulin sensitivity in diet-induced obese rats.

We noted that there was no increase in serum insulin concentrations in HFD-fed mice with insulin resistance. The lack of such an increase indicates the pancreatic beta cells were not responding by increasing insulin secretion, even though the animals may remain insulin resistant. The mechanisms underlying the uncoupling of insulin resistance and elevated plasma insulin warrants further investigation.

Intriguingly, in our study, annatto-extracted TT was as effective as metformin in lowering fasting blood glucose levels and improving glucose tolerance. Analysis of the pancreas by immunostaining revealed no gross morphological changes in the alpha (as demonstrated by glucagon staining) or beta (as demonstrated by insulin staining) islet cells between any of the treatment (the HFD, T400, T1600 and Met) groups and the LFD group (Fig. 2). Consistently, TUNEL analysis of the pancreas from LFD and HFD groups showed no differences in the number of apoptotic cells. Hyperinsulinemia was not observed in any of the mice at the time of sample collection. The lack of elevated serum and pancreatic insulin levels in the fasting HFD mice along with no gross morphological changes in the islets, despite an increase in blood glucose levels, suggests possible beta cell dysfunction rather than beta cell loss as the islets should respond to elevated blood glucose by increasing insulin production and secretion. Prolonged exposure to hyperglycemia and hyperlipidemia can result in beta cell glucotoxicity and lipotoxicity^34^. Confirmation of beta cell dysfunction will require further analysis in future studies.

Metformin, a commonly used drug for the initial treatment of patients with T2DM (American Diabetes Association), was shown to benefit bone metabolism^35–37^. Metformin increases trabecular bone formation through activation of AMPK signaling, a major intracellular pathway that senses energy starvation, in osteoblastic cells^36^. Metformin also promotes osteoblast differentiation and inhibits differentiation of stem cells into adipocytes through an inhibition of PPARγ, a nuclear receptor that regulates lipid and glucose metabolism^35^. Li *et al*.^37^ reported that metformin re-balances catabolism and nitrogen disposal and improves bone homeostasis in animal models of hyperglycemia. Moreover, male diabetic patients taking metformin had a significant reduction of serum bone resorption^36^. In this study, we compared the impact of metformin treatment and annatto-TT supplementation on glucose homeostasis, bone turnover biomarkers, and bone microstructure. It was observed that annatto-TT has an equal (the T400 group = the Met group) or even more favorable impact (the T1600 group> the Met group) on ITT AUC, serum P1NP, Tb.Th, Conn. Dn, Tb.Sp, SMI, and Ct.Th, suggesting TT-supplementation may be an alternative to Met for controlling hyperglycemia and providing bone benefits simultaneously. Such findings corroborate our previous finding that anti-diabetic supplementation, Yunzhi with anti-oxidant and anti-inflammatory characteristics, improved bone properties in diabetic rats, in part, by improvement in hyperglycemic control in STZ-induced DM animals^7^.

Inflammation may be the most important mechanism linking obesity to the development of T2DM^38^. Emerging evidence has suggested that in the development of obesity, greater adipose tissue mass directly contributes to systemic inflammation^39,40^ and chronic oxidative stress^41^ that are detrimental to bone^28,42–44^. T2DM-related systemic chronic inflammation can elevate bone inflammatory cells and mediators, leading to an inhibition of osteoblast markers^45,46^. Obesity and HFD have been shown to increase the supply of free fatty acids in the liver, resulting in hepatic lipid accumulation and inflammation via NF-κB activation and cytokine production^26,47^. In obese animals, the chemotactic signals originating from adipose tissue and liver result in recruitment of monocytes to the liver and their subsequent differentiation into proinflammatory macrophages^48^. Thus, the hepatic inflammatory response to obesity is perpetuated by both resident Kupffer cells and newly acquired macrophages^48^. Indeed, we found that the HFD-fed mice with greater body weight and hyperglycemia had elevated liver mRNA expression of inflammatory cytokines^26^. We also found an inverse relationship between liver mRNA expression of pro-inflammatory cytokines and bone volume at trabecular bone as well as cortical thickness at midshaft bone in the HFD-fed rats^49^. Supplementation of annatto-TT appeared to reverse the effect of HFD on inflammation and bone microstructure, suggesting TT’s osteo-protective function is mediated through a suppression of inflammation.

The increased expression of *Itgax* (CD11c) and *Adgrel* (F4/80) in the livers of HFD-fed mice indicates the increased presence of immune cells, likely inflammatory (M1) macrophages. The HFD-fed mice also had increased inflammatory cytokine expression in the liver, and TT normalized the expression of these inflammatory cytokines to levels comparable to LFD-fed mice (Table 2). These data indicate that TT prevents HFD-induced hepatic inflammation, despite having no effect on body weight. Our previous work found that treating HFD-fed mice with TT decreased adipose tissue inflammation, adipocyte size, and macrophage infiltration in the adipose tissue^26^. Additionally, we reported decreased hepatic steatosis and serum triglyceride levels and reduced macrophage infiltration in the liver^26^. Moreover, 4 weeks of γ-TT treatment decreases HFD-induced systemic and adipose tissue inflammation^50^. It is well known that adipose tissue inflammation is strongly linked to hepatic inflammation. Thus, it is possible that the anti-inflammatory effects of TT in adipose tissue lead to secondary reductions in hepatic inflammation, although direct anti-inflammatory effects in hepatocytes^51^ and in the heart^33^ were previously reported.

The 400 and 1600 mg/kg diet doses of TT for mice are equivalent to daily doses of 160 mg and 640 mg, respectively, for a 70-kg human adult, based on body surface areas of mice and humans^52^. Since TT can only be found in certain plants, e.g., annatto, palm, grains, nuts, and rubber^17,53^, such doses in humans are more likely obtainable through supplements rather than diet.

We recently reported a mechanism of bone protection by TT through the enhancement of antioxidant capacities and the suppression of oxidative stress and inflammation^54^.

In the present investigation, we corroborated the ability of annatto-extracted TT, a generally recognized as safe (GRAS) dietary supplement, to improve glucose homeostasis and mitigate the deterioration of bone microarchitecture in obese male mice. Our data demonstrate that supplementation of TT in a HFD has anti-diabetic effects by improving glucose intolerance and insulin intolerance, and also has osteo-protective effects by conserving bone matrix and microarchitecture in HFD-induced obese mice with hyperglycemia and insulin resistance. These osteoprotective effects may be mediated in part through favored bone turnover markers as well as bone remodeling along with modulated cancellous and endocortical bone compartments, resulting in a larger net bone matrix.

The primary goal of this study was to determine whether TT improves glucose homeostasis and mitigates bone deterioration in obesity/diabetes through suppression of inflammation. Therefore, the most relevant model of human obesity/diabetes is the high-fat diet-induced obese mice with elevated inflammation, insulin resistance, and glucose tolerance. However, it would be interesting to also know the benefits of TT in lean or low fat fed models without chronic inflammation or altered glucose metabolism, and we hope to conduct such studies in the future.

We noted that histological data showing decreased inflammation in bone and liver, and changes in osteoclasts/osteoblasts would have further supported the mechanisms of action. We previously reported that both T400 and T1600 reduce inflammation in the liver histologically^26^. In the present study, as an alternative to histology, we demonstrated that both T400 and T1600 groups mitigated HFD-induced bone microstructure deterioration (Table 2). In future studies, we should include such histologial findings to support the mechanisms of action due to TT. Furthermore, it is also worthy to investigate the effect of TT on functional parameters to show if the increased risk of fracture in osteoporotic bones can be alleviated by TT treatment.

In summary, in the present study, annatto-extracted TT was assessed as an alternative dietary supplement for bone health in obese mice with glucose intolerance and insulin intolerance. Our data show that annatto-extracted TT has anti-diabetic and bone-protective effects in terms of glucose homeostasis, bone turnover biomarkers, bone volume, and bone microarchitecture in obese mice through, in part, a suppression of liver inflammation. The current animal study represents a critical step towards understanding the impact of annatto-extracted TT on bone health in obese T2DM patients. Given the millions of individuals afflicted with diabetes or prediabetes who are also at risk for devleping osteoporosis, the results of this study could have a significant impact on future treatment strategies. Future studies based on the current animal study are warranted to address the differences between obese T2DM male mice and obese T2DM patients and the translational value of annatto-extracted TT in glucose homeostasis management and bone health.

## Materials and Methods

### Animals and treatments

Fifty-eight male C57BL/6 J mice (5-week-old, Jackson Laboratory, Bar Harbor, ME, USA) were fed chow diet and distilled water ad libitum for 5 days. After acclimatization, mice were weighed, randomly stratified by weight and assigned to 5 groups: low-fat diet group (LFD, 5% of energy from fat), HFD group (58% of energy from fat), HFD + 400 mg TT/kg diet (T400), HFD + 1600 mg TT/kg diet (T1600), and HFD + 200 mg metformin/kg diet (Met, used as a positive control treatment for T2DM) for 14 weeks. Animals had free access to water and diet during the study period. TT (gift from American River Nutrition, Inc., Hadley, MA) was extracted from annatto oil and 70% pure, containing 90% δ -TT and 10% γ -TT. TT was premixed with tocopherol-stripped soybean oil (Dytes Inc., Bethlehem, PA) before being added to HFD. Previous animal studies showed the osteoprotective impacts of delta-tocotrienol at 60 mg/kg body weight in rats^21,24^. Our T400 and T1600 mg/kg diet in mice corresponds to 28 mg/kg body weight and 112 mg/kg body weight in rats, respectively, which is comparable with the previous published works^21,24^.

Mice were housed in cages of three and maintained at a controlled temperature of 21±2 °C with a 12 h light-dark cycle. Each treatment group had 4 cages of mice. All animals were observed daily for clinical signs of disease. Body weight, food intake, and water consumption were recorded weekly. All conditions and handling of the animals were approved by the Texas Tech University Health Sciences Center Institutional Animal Care and Use Committee. All experiments were performed in accordance with the relevant guidelines and regulations.

### *In vivo* glucose and insulin tolerance tests

Intraperitoneal GTT was performed after 12 weeks of treatment. Glucose (2 mg/g body weight) was injected intraperitoneally into mice (n > 6 per treatment group) fasted for 4 h. Blood samples for glucose determination were obtained prior to (0 mins) and at 15, 30, 60, and 120 mins following glucose administration. For intraperitoneal ITT, after 12 weeks of treatment, mice (n = 6 per treatment group) were fasted for 4 h prior to intraperitoneal injection of insulin (Humulin, Abbott, Chicago, IL) at 1 U/kg body weight. Blood glucose was measured prior to (0 mins) and at 15, 30, 60, and 120 mins following insulin injection. The AUC was calculated by trapezoidal method.

### Sample collection

At the end of the experiment, animals were fasted for 4-hours and blood was collected from mice anesthetized with isoflurane. Femur and lumbar vertebrae-4 (LV-4) were harvested and cleaned of adhering soft tissues, and stored in 70% ethanol at 4 °C for later analyses. Blood samples were centrifuged at 1500 × g for 20 minutes and the serum samples were obtained and kept at −80° until analyzed.

### Analysis of pancreatic tissue

At the end of the experiment, pancreases (n = 4 per treatment group) were fixed in Z-fix and paraffin embedded for histological assessment. Tissue sections were immunostained for insulin and glucagon as described previously^55^. Primary antibodies included guinea pig anti-insulin (diluted 1:1000; Dako Agilent Pathology Solutions, Santa Clara, CA) and mouse anti-glucagon (diluted 1:5000; Sigma). Biotinylated secondary antibodies and avidin–biotin–enzyme complexes were purchased from Vector Laboratories (Burlingame, CA). Diaminobenzidine was used as a chromogen (BioGenex, Fermont, CA). All sections were counterstained with hematoxylin. Apoptosis was determined using the DeadEnd^TM^ Fluorometric TUNEL assay (Apoptosis Detection System; Promega Corporation, Madison, WI) according to the manufacturer’s instructions. Negative controls included sections incubated without the TdT enzyme. For quantification, the total number of apoptotic cells per tissue section was counted and normalized to the total tissue area.

### Serum biomarkers analyses

The concentrations of P1NP and CTX were determined in serum using the respective kits (Immunodiagnostic System Ltd, Scottsdale, AZ, USA). Intra-assay and inter-assay coefficients of variance were 5.0% and 8.0% for P1NP as well as 5.6% and 10.5% for CTX, respectively.

### Bone microarchitecture measurement with μ-CT

The LV-4 and right femur were scanned using micro-computed tomography (μCT) (Model: Scanco μCT 40; SCANCO Medical AG, Switzerland) following the procedures of Cao *et al*.^49^ and the recommended guidelines for μCT scanning^56^. Bone nomenclature was based on Parfitt *et al*.^57^ Trabecular parameters in both LV-4 and femur included BV/TV, Tb.N, Tb.Th, Tb.Sp, Conn.Dn, and SMI. Cortical parameters in femur included B.Ar, T.Ar, and Ct.Th.

### Measurement of mRNA expression in liver

RNA was extracted from liver samples using an RNeasy mini kit (QIAGEN, Valencia, CA). On-column DNA digestion was performed using QIAGEN’s RNase-free DNase set. RNA was quantified using the Nanodrop ND 1000 Spectrophotometer. 2 µg RNA was reverse transcribed to produce cDNA. Gene expression was measured by real-time PCR (Applied Biosystems, Foster City, CA) by measurement of SYBR Green. mRNA levels were normalized to cyclophilin expression, and were analyzed using the 2^−ΔΔCT^ method. Cyclophilin expression levels were unchanged in response to diet or treatment group. Primer sequences are as follows: ***Ccl2*** (MCP-1) Fwd 5′- GGAGAGACAGCGGTCGTAAG-3′; Rev 5′-CCAGCCGGCAACTGTGA-3′; ***Emr1*** (F4/80) Fwd 5′-GTGCCATCATTGCGGGATTC-3′; Rev 5′- GACGGTTGAGCAGACAGTGA-3′; ***Il2*** Fwd 5′-CATGCAGCTCGCATCCTGT-3′; Rev 5′-AAGTGGGGCTTGAAGTGGG-3′; ***Il23*** Fwd 5′-CTTGCCCTTCACGCAAAACA-3′; Rev 5′- CAAAGGATCCGCCAAGGTCT-3′. ***Ifng*** Fwd 5′-GAGGTCAACAACCCACAGGT-3′; Rev 5′-GGGACAATCTCTTCCCCACC-3′; ***Itgax*** Fwd 5′-GGGACGCTTACCTGGGTTAC-3′; Rev 5′-CCTGGAAATCTCTGCAGGTGT-3′; ***Ppia*** (Cyclophilin) Fwd 5′-CTTCGAGCTGTTTGCAGACAAAGT-3′; Rev 5′-AGATGCCAGGACCTGTATGCT-3′.

### Statistical analysis

Results were presented as mean ± standard error of the mean (SEM). For TUNEL quantification, significant differences in means were determined by the unpaired Student’s t-test method. All the other data were tested by one-way analysis of variance (ANOVA) followed by Fisher’s Least Significant Difference (LSD) *post hoc* test with SigmaStat software, version 12.5 (Systat Software, Inc., San Jose, CA). A significance level of p < 0.05 applies to all statistical tests.
